# Reply to Stohner: On the significance of BMI-age dependence of exhaled aerosol

**DOI:** 10.1073/pnas.2107559118

**Published:** 2021-06-29

**Authors:** David A. Edwards, Jonathan Salzman, Robert Langer

**Affiliations:** ^a^John A. Paulson School of Engineering & Applied Sciences, Harvard University, Cambridge, MA 02139;; ^b^R&D Department, Sensory Cloud, Boston, MA 02142;; ^c^Department of Chemical and Biological Engineering, Massachusetts Institute of Technology, Cambridge, MA 02138

Stohner (1) mistakenly identifies the correlation drawn in our recent article (2) between exhaled aerosol and body mass index (BMI)-age as based on a figure legend to which the article does not itself allude. In our article (2), we establish that those individuals with high BMI-years (above 650) exhaled significantly more aerosol than those with low BMI-years (below 650). Those with BMI-years less than 650 exhaled a mean of 29.81 ± 9.83, and those with BMI-years above 650 exhaled a mean of 183.32 ± 84.15. A two-tailed *t* test reveals significance (*P* = 0.0006)—as is pointed out in our article (2). Stohner ignores the BMI-age assessment of significance made in the article and bases his argument of no correlation on a reference to the legend of figure 2 in ref. 2 that refers to a regression analysis with *r*^2^ = 0.98. We do not claim, in ref. 2, that the relation between exhaled aerosol and BMI-years is linear—obviously, this is not true, based on visual inspection of the data. Unfortunately, the legend to figure 2 in ref. 2 was prepared for an earlier version of the manuscript and not updated in the final submission. Stohner informed us of the oversight on March 30, leading us to submit an amended legend (see ref. 2 correction) to the editors immediately thereafter and inform Stohner. Stohner also argues we might have presented our exhaled aerosol other than from the highest to the lowest emitters of aerosol. Presenting exhaled aerosol data as we do in figure 1 in ref. 2, from highest to lowest exhaled aerosol number, illustrates that individuals in the study exhaled a continuum of respiratory droplet numbers across several orders of magnitude, as appears to be common to large groups of human subjects (3, 4). Our distinction, in the article, of two groups, superspreaders and low spreaders (of exhaled aerosol), is based on the observation that ∼20% of the individuals exhaled ∼80% of the aerosol—a classical superspreader distribution of airborne infectious disease (5). We believe the identification of the 20% highest aerosol-emitting individuals as a superemitting group is noteworthy in light of the present scientific debate (6) around the role exhaled respiratory droplets play in the spread of contagion.
